# Benefits of non-work interactions with your supervisor: Exploring the bottom-up effect of employee boundary blurring behavior on abusive supervision

**DOI:** 10.3389/fpsyg.2022.941990

**Published:** 2022-09-29

**Authors:** Luyuan Jiang, Guohua He, Hansen Zhou, Laijie Yang, Xiaolan Li, Wenpu Li, Xin Qin

**Affiliations:** ^1^Sun Yat-sen Business School, Sun Yat-sen University, Guangzhou, China; ^2^School of Economics and Management, Tsinghua University, Beijing, China

**Keywords:** abusive supervision, boundary blurring, liking, bottom-up effect, self-disclosure theory

## Abstract

Abusive supervision has long been found to have remarkably negative impacts on individual and organizational outcomes. Accordingly, prior studies have explored many organizational and supervisory predictors of abusive supervision and offered several interventions to reduce it. However, extant research lacks the bottom-up perspective to explore how employees can act to reduce abusive supervision, which is an important factor that enriches abusive supervision literature and helps employees protect themselves from being abused. Drawing on self-disclosure theory, we develop a model of whether and how employee boundary blurring behavior may protect them from being abused by their supervisors. Specifically, we conducted two studies to test the theoretical model, including a scenario-based experimental study and a multi-source, multi-wave field study. The results reveal a negative indirect effect of employee boundary blurring behavior on abusive supervision *via* supervisor liking toward the employee. By uncovering employee boundary blurring behavior as an antecedent of abusive supervision, we enrich the abusive supervision literature with a bottom-up behavioral strategy for employees to proactively protect themselves from being abused. We hope our findings will encourage future studies to identify boundary conditions and other solutions for employees to minimize the risk of being abused.

## Introduction

Abusive supervision, defined as “the extent to which supervisors engage in the sustained display of hostile verbal and non-verbal behavior, excluding physical contact” toward employees (Tepper, 2000, p. 178), has long been found to wreak havoc far and wide (for reviews, see Tepper, 2007; Martinko et al., 2011; Mackey et al., 2017). For example, abusive supervision was found to result in employees’ decreased performance and well-being, and increased misbehaviors and turnover (Detert et al., 2007; Tepper, 2007; Walter et al., 2015). Accordingly, scholarly interest has grown in identifying the antecedents of abusive supervision, explaining when and how supervisors may engage in such behavior, and thus offering effective interventions to reduce it (Zhang and Bednall, 2016; Tepper et al., 2017; Qin et al., 2018a). Specifically, existing studies have mainly focused on how contextual and supervisory factors may explain abusive supervision (Zhang and Bednall, 2016; Tepper et al., 2017). For contextual predictors, many research has found that aggressive organizational norms, abusive role model, and power distance are positively associated with abusive supervision (Restubog et al., 2011; Vogel et al., 2015). For supervisory predictors, many research found that supervisors’ characteristics (e.g., Machiavellian and the dissimilarity of demographics between supervisors and employees), stress and negative states (e.g., lack of sleep and negative emotions) may lead them to engage in abusive supervision because of identity threat and self-regulation impairment (Kiazad et al., 2010; Tepper et al., 2011; Mawritz et al., 2014; Barnes et al., 2015). Previous studies have thus provided valuable insights on how abusive supervision can be reduced by identifying contextual and supervisory factors.

However, we still have relatively limited knowledge about the antecedents of abusive supervision from the employee’s perspective (for an exception, see Huang et al., 2022). The scarce relevant studies mainly focus on when and how certain characteristics of employees (e.g., narcissism and negative affectivity) make them more likely to be victims of abusive supervision (Lian et al., 2012; Harvey et al., 2014; Henle and Gross, 2014; Chen et al., 2021b). Given the relative stability of employee characteristics offers little scope to reduce abusive supervision, these studies offer limited help in understanding how employee can proactively avoid being abused. Therefore, existing studies on the antecedents of abusive supervision generally ignore the possibility that employees may engage in proactive behaviors to protect themselves from being abused.

In this research, we examine whether and how employee boundary blurring behavior—the act of blurring the boundary between professional and personal life domains (Rothbard et al., 2022)—affects abusive supervision. Exploring this potential relationship is theoretically important because it enriches abusive supervision literature by identifying employee boundary blurring behavior as a possible antecedent of abusive supervision from the employee’s perspective, which has traditionally been overlooked in the literature. Practically, knowledge of the potential benefits of employee boundary blurring behavior can be leveraged to help employees avoid being abused, which would, in turn, improve their performance and well-being (Tepper, 2000; Lin et al., 2013; Zhang and Bednall, 2016). Thus, our study provides a more comprehensive understanding of antecedents of abusive supervision from a bottom-up perspective, which may help employees effectively protect themselves from being abused.

To address these questions, we draw on self-disclosure theory (Cozby, 1973; Collins and Miller, 1994) and propose that employee boundary blurring behavior may increase supervisor liking toward the employee, thereby reducing abusive supervision. Self-disclosure theory posits that self-disclosure induces interpersonal attraction and shapes relationship between people (Cozby, 1973; Collins and Miller, 1994; Sprecher et al., 2013). According to this theory, employee boundary blurring behavior (e.g., sharing personal issues in the workplace) is a form of self-disclosure that may make a supervisor more inclined to like an employee by fostering the perception that the employees desire to establish a more intimate relationship through such behavior (Collins and Miller, 1994; Nifadkar et al., 2019; Rothbard et al., 2022). In turn, when a supervisor finds the employees more likable, they tend to perceive less threat and hostility toward them, and thus restrain his or her abusive tendencies (Tepper et al., 2011; Chen et al., 2021a).

**Figure 1:**
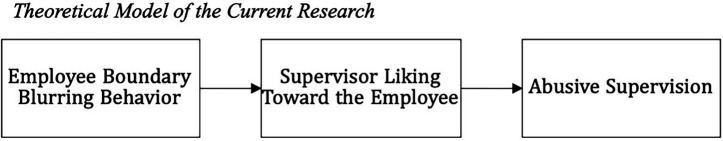
Theoretical model of the current research.

To test our theoretical model (see Figure 1), we conducted two studies: an experimental study and a multi-source, multi-wave field study. Our research makes several contributions to abusive supervision literature and boundary management literature. First, we contribute to the abusive supervision literature by investigating employee boundary blurring behavior as a bottom-up behavioral strategy for employees to proactively protect themselves from being abused. Prior studies on the antecedents of abusive supervision have primarily focused on the contextual and supervisory predictors (e.g., Kiazad et al., 2010; Restubog et al., 2011; Tepper et al., 2011; Liu et al., 2012; Mawritz et al., 2012; Barnes et al., 2015). However, these studies have overlooked another important perspective, namely a bottom-up perspective, to explore how employees can act proactively to avoid being abused. Therefore, our study addresses the above research gap by investigating the effect of employee boundary blurring behavior on abusive supervision. Second, we extend the boundary management literature by exploring the potential positive outcomes of employee boundary blurring behavior in the workplace. Previous studies have focused on individuals’ management strategies of the boundaries between work and family and the effects of different strategies on their work-family relationships (Ashforth et al., 2000; Kreiner et al., 2009; Allen et al., 2014). Our study contributed to the boundary management literature by examining the impact of employee boundary blurring behavior on one kind of supervisor behavior toward the employee.

## Theoretical grounding and hypothesis development

### Employee boundary blurring behavior and supervisor liking toward the employee

Self-disclosure theory suggests that by disclosing personal information, an individual affects others’ feeling of interpersonal attraction toward and subsequent interactions with that person (Cozby, 1973; Collins and Miller, 1994). Specifically, during the development of interpersonal relationships, the disclosure of detailed or even private information increases the level of intimacy (Clark and Reis, 1988; Collins and Miller, 1994). In the workplace, high interpersonal attraction between supervisor and employee is associated with increased trust, supportive behaviors, and employee performance (Reb et al., 2019; Parent-Rocheleau et al., 2020).

Drawing on self-disclosure theory, we posit that supervisors tend to show a high level of liking for employees who engage in boundary blurring behavior—acts of blurring the boundary between professional and personal life domains (Rothbard et al., 2022), such as by publicly displaying family pictures in the office, attending company parties, and sharing non-work information in the workplace (Byron and Laurence, 2015; Nifadkar et al., 2019). Employees could manage their self-disclosure toward their supervisors by controlling whether and how to engage in boundary blurring behavior with them. In doing so, employees may facilitate several dynamics to foster supervisors’ positive feelings toward them (Phillips et al., 2009; Rothbard et al., 2022). First, since self-disclosure is considered as social exchange within ongoing relationships (Collins and Miller, 1994), supervisors tend to reward employees who engage in boundary blurring behavior with a higher level of liking, because they may perceive those employees are endeavoring to establish a more intimate relationship (Worthy et al., 1969; Archer and Cook, 1986; Sprecher et al., 2013; Lin and Utz, 2017; Yang, 2020). Second, as self-disclosure may shape others’ perceptions of the discloser, supervisors may regard employees who blur boundaries as warmer and more responsive (Laurenceau et al., 1998; Rothbard et al., 2022). Furthermore, as people usually tend to segment their professional and personal life for fear of damaging their professional reputation (Kreiner et al., 2006), employees who voluntarily engage in boundary blurring behaviors may be perceived by supervisors as more confident, sincere, and authentic. Existing empirical research provides some support for this prediction. For example, recent studies suggest that employee boundary blurring behavior in the workplace, such as displaying family pictures and discussing non-work matters, helps build closer relationships with coworkers (Dumas et al., 2013; Byron and Laurence, 2015; Whitman and Mandeville, 2021). These findings further suggest that boundary blurring behavior may facilitate interpersonal liking. Thus, we propose the following:

*Hypothesis 1*: Employee boundary blurring behavior is positively related to supervisor liking toward the employee.

### The effect of supervisor liking toward the employee on abusive supervision

We further propose that when a supervisor likes an employee, they are less likely to engage in abusive supervision. First, supervisors who like their employees are more inclined to view them favorably, view them in a more positive light (Regan et al., 1974; Dulebohn et al., 2017), and less likely to categorize them as provocations of hostility and threat, which have both been proven to be important predictors of abusive supervision (Tepper et al., 2012; Mawritz et al., 2017; Eissa et al., 2020; Li et al., 2020; Chen et al., 2021a). Second, the literature on interpersonal attraction indicates that supervisors tend to build high-quality relationships with employees they like (Engle and Lord, 1997), which may motivate them to exercise high ethical standards in their treatment of these employees (Tepper et al., 2011; Walter et al., 2015), and thus restrain their abusive intentions. Extant empirical research also supports the negative relationship between supervisor liking toward the employee and abusive supervision. For example, supervisors are less likely to abuse employees toward whom they have interpersonal attraction, such as similarity attraction (Tepper et al., 2011). Taken together with Hypothesis 1, we propose the following:

*Hypothesis 2*: Supervisor liking toward the employee is negatively related to abusive supervision.

*Hypothesis 3*: Employee boundary blurring behavior has an indirect effect on abusive supervision *via* supervisor liking toward the employee.

### Overview of current research

To test our theoretical model, we conducted an experimental study and a multi-source, multi-wave field study. Specifically, in Study 1, we conducted a scenario-based experiment, where we asked participants to act as team leaders while manipulating employee boundary blurring behavior. Through the experiment, we are able to establish the causal relationship between employee boundary blurring behavior and abusive supervision. To maximize external validity, in Study 2, we conducted a 3-wave multi-source survey in a large manufacturing company and measured all the variables in our theoretical model. Thus, our multimethod research design (i.e., an experimental study and a field survey) provides evidence for high internal and external validity of our findings.

## Study 1 method

### Participants

We recruited 175 participants with supervisory responsibilities from the United States *via* Prolific, a widely used online survey platform proven to supply diverse and attentive respondents (Palan and Schitter, 2018). This sample size ensured a power level of 0.90 to detect a medium effect (*f =* 0.25), assuming an *α* level of 0.05 (Faul et al., 2009). Each participant received USD 0.4 as compensation. Following the recommendation of Meade and Craig (2012), we included an attention-check item in the questionnaire. Among the sample, 60.0% were female, 78.3% were Caucasian, the average age was 37.3 years old (*SD* = 11.4), and average education was 15.9 years (*SD* = 2.3). Participants worked in various industries, including healthcare (17.7%), education (12.0%), service (11.4%), retailing (10.9%), IT (10.3%), and others (37.7%). They were also from different departments, including administration (34.9%), technology (26.3%), finance (7.4%), marketing (4.6%), and others (26.8%).

### Procedures and experiment design

We conducted a between-subject experiment and manipulated employee boundary blurring behavior. Specifically, we generated two experimental conditions: high vs. low employee boundary blurring behavior and randomly assigned our participants to one of them. Following the critical incident technique paradigm (Aquino et al., 2001), all participants were instructed to visualize themselves in the roles of team leaders. In each condition, participants were instructed to read a scenario describing an employee who either engages in boundary blurring behavior (*n* = 87) or avoids doing so (*n* = 88). In line with prior studies (e.g., Watkins et al., 2019), they were then presented with a managerial situation. Following the scenario descriptions and managerial situation, participants were required to complete the questionnaires measuring supervisor liking toward the employee and abusive supervision intention, respond to the manipulation-check items, and report their demographic information.

#### Manipulation of employee boundary blurring behavior

To manipulate employee boundary blurring behavior, participants were instructed to put themselves in the role of team leaders, reading a statement describing boundary blurring behaviors that employees exhibit toward their leaders (for similar research design, see Watkins et al., 2019). Specifically, in the high employee boundary blurring behavior condition, participants read the following scenario:

You have been working in a manufacturing company. Your current position is a team leader. Alex is one of your direct reports who has an average performance. You have noticed that Alex strives to build a personal connection with you. Specifically, Alex usually interacts with you on Facebook, talks with you about his/her personal life, and goes to some social activities with you (e.g., sporting events, after-work drinks).

In the low employee boundary blurring behavior condition, participants read another scenario:

You have been working in a manufacturing company. Your current position is a team leader. Alex is one of your direct reports who has an average performance. You have noticed that Alex tends to avoid building a personal connection with you. Specifically, Alex seldom interacts with you on Facebook, never talks with you about his/her personal life, and refuses to go to any social activities with you (e.g., sporting events, after-work drinks).

At the end of each scenario, participants in both conditions read the following managerial situation:

Recently, you have been chosen to lead a large project, which is very important to your team and yourself. You don’t want anyone to mess it up. You have given Alex some important assignments of this project. However, you find that Alex made a serious mistake, which may lead the project to be stopped, or even worse.

### Measures

Unless otherwise specified, all measures for the two studies used a five-point Likert-type scale ranging from 1 = “Strongly disagree” to 5 = “Strongly agree.” All items are presented in Appendix A.

#### Supervisor liking toward the employee

Supervisor liking toward the employee was measured using Hamstra et al.’s (2013) four-item scale. Sample items are “This employee seems like a pleasant person to me” and “I think it is pleasant to work with this employee” (*α* = 0.90).

#### Abusive supervision intention

Abusive supervision intention was measured using Mitchell and Ambrose’s (2007) five-item scale adapted from Tepper (2000), which has been widely adopted by previous research (e.g., Wee et al., 2017; Qin et al., 2018a; Chen et al., 2021a). Participants rated their likelihood of engaging in abusive supervisory behavior toward the employee, responding on a five-point Likert-type scale ranging from 1 = “Extremely unlikely” to 5 = “Extremely likely.” Sample items are “To ridicule him/her” and “To put him/her down in front of others” (*α* = 0.83).

#### Manipulation check

To test the effectiveness of our manipulation of employee boundary blurring behavior, we used a four-item scale from Rothbard et al.’s (2022) boundary blurring activities scale. Sample items are “This employee would like to connect with me on Facebook” and “This employee would like to talk with me about his/her personal life during work hours” (*α* = 0.98).

### Analytic strategy

To test our hypotheses, we conducted two-sample *t*-tests by condition and ordinary least squares (OLS) regressions. To test the indirect effect in Hypothesis 3, we also employed RMediation (Tofighi and MacKinnon, 2011), which estimates Type I error rates more accurately than traditional mediation tests, such as the Sobel test (MacKinnon et al., 2007).

## Study 1 results

### Manipulation check

We first conducted a *t*-test to examine whether our manipulation of employee boundary blurring behavior was effective. The results showed that employee boundary blurring behavior was perceived to be significantly higher in the high employee boundary blurring behavior condition (*M* = 4.30, *SD* = 0.56) than in the low condition (*M* = 1.50, *SD* = 0.66), *t* (173) = −30.25, *p* < 0.001, *d* = −4.58, thus indicating that our manipulation was successful.

### Tests of hypotheses

Descriptive statistics and correlations are reported in Table 1. Hypothesis 1 posits that employee boundary blurring behavior is positively related to supervisor liking toward the employee. The *t*-test results revealed that participants in the high employee boundary blurring behavior condition reported a significantly higher level of supervisor liking toward the employee (*M* = 3.48, *SD* = 0.69) than did participants in the low employee boundary blurring behavior condition (*M* = 2.49, *SD* = 0.73), *t* (173) = −9.30, *p* < 0.001, *d* = −1.41. The OLS regression results also showed that employee boundary blurring behavior was significantly positively related to supervisor liking toward the employee (Model 1, Table 2; *b* = 1.00, *SE* = 0.11, *p < 0*.001). Thus, Hypothesis 1 was supported in Study 1.

**Table 1:** Descriptive statistics and correlation coefficients of variables in Study 1.

Variables	Mean	*SD*	1	2
1. Employee boundary blurring behavior	0.50	0.50		
2. Supervisor liking toward the employee	2.98	0.87	0.58^***^	
3. Abuse supervision intention	1.48	0.58	−0.20^**^	−0.34^***^

*n* = 175. *n* = 88 in low boundary employee blurring behavior condition; *n* = 87 in high boundary employee blurring behavior condition. For employee boundary blurring behavior, 0 = low boundary employee blurring behavior condition, 1 = high employee boundary blurring behavior condition.

^*^p < 0.05; ^**^p < 0.01; ^***^p < 0.001.

**Table 2:** The effects of employee boundary blurring behavior and supervisor liking toward the employee on abusive supervision in Study 1.

Variables	Supervisor liking toward the employee			abusive supervision intention
	Model 1			Model 2			Model 3		
	*b*	*SE*	*t*	*b*	*SE*	*t*	*b*	*SE*	*t*
Employee boundary blurring behavior	1.00	0.11	9.31^***^	−0.24	0.09	−2.74^**^	−0.01	0.10	−0.11
Supervisor liking toward the employee							−0.23	0.06	−3.82^***^
Constant	2.49	0.08	32.93^***^	1.60	0.06	26.14^***^	2.16	0.16	13.62^***^
*R^2^*	0.33^***^			0.04^**^			0.12^***^		

*n* = 175. *n* = 88 in low boundary employee blurring behavior condition; *n* = 87 in high boundary employee blurring behavior condition. For boundary blurring behavior, 0 = low boundary blurring behavior condition, 1 = high boundary blurring behavior condition.

^*^p < 0.05; ^**^p < 0.01; ^***^p < 0.001.

Hypothesis 2 proposes that supervisor liking toward the employee is negatively related to abusive supervision. As shown in Model 3 of Table 2, supervisor liking toward the employee was significantly negatively related to abusive supervision (*b* = −0.23, *SE* = 0.06, *p* < 0.001). Thus, Hypothesis 2 was supported in Study 1.

Hypothesis 3 proposes that employee boundary blurring behavior has an indirect effect on abusive supervision *via* supervisor liking toward the employee. The *t*-test results revealed that participants in the high employee boundary blurring behavior condition had significantly lower intention to engage in abusive supervision (*M* = 1.36, *SD* = 0.46) than did those in the low employee boundary blurring behavior condition (*M* = 1.60, *SD* = 0.66), *t* (173) = 2.75, *p* = 0.007, *d* = 0.41. The OLS regression results also showed that employee boundary blurring behavior was significantly negatively related to abusive supervision (Model 2, Table 2; *b* = −0.24, *SE* = 0.09, *p = 0*.007). We then used RMediation to test the indirect effect of employee boundary blurring behavior on abusive supervision *via* supervisor liking toward the employee. The results revealed that the indirect effect was significantly negative (estimate = −0.23, *SE* = 0.07, 95% CI = −0.34, −0.13). Thus, Hypothesis 3 was supported in Study 1.

Overall, the results of Study 1 provide preliminary support for the indirect effect of employee boundary blurring behavior on abusive supervision *via* supervisor liking toward the employee. Although the experiment provides causal support for our theoretical model, it is necessary to investigate whether the observed effects also exist in a real organizational context. Hence, we conducted Study 2 to test our model in an actual organizational setting, thereby assessing its external validity.

## Study 2 method

### Participants and procedures

To further test our hypotheses, we collected multi-source data in three waves from a large manufacturing company in Southern China, which operates one of the country’s biggest plants specializing in the precision production of high-end furniture parts. We first contacted the company’s human resources (HR) director to reach out for supervisors and employees. After getting permission and consents, we invited 56 supervisors and their direct reports (*n* = 311) to participate in this research. All supervisor and subordinate respondents were assured of the confidentiality of their responses. We then invited supervisors to respond to questionnaires about their subordinates as well as themselves during the first two rounds of survey, while inviting their immediate subordinates to participate in the third round of survey. Identification codes were used to match supervisor-employee responses across the three waves. Each supervisor was compensated with RMB 35 (approximately USD 5.5), while each employee received RMB 10 (approximately USD 1.5).

Each wave had a one-week interval. At Time 1 (T1), we asked supervisors to rate employee boundary burring behavior and report their demographic information; 55 supervisors responded (98.2% response rate), rating 305 employees. At Time 2 (T2), we sent a second survey link to supervisors who had completed the T1 survey, asking them to rate their liking of each employee: 52 supervisors responded (94.6% response rate), rating 255 employees. At Time 3 (T3), we sent a third survey link to 250 employees who had been assessed by their supervisors at both T1 and T2. Employees were asked to rate their supervisors’ abusive supervision; 246 employees responded (98.4% response rate).

After matching data from the three waves for supervisors and employees, we obtained a final sample of 49 supervisors (87.5% final response rate) and 216 employees (69.5% final response rate). In the final supervisor sample, 81.6% were male, the average age was 32.3 years (*SD* = 7.7), and average education was 11.6 years (*SD* = 2.8). In the final employee sample, 52.8% were male, the average age was 32.3 years (*SD* = 8.2), and average education was 10.6 years (*SD* = 3.0). The average dyadic tenure between supervisors and employees was 2.0 years (*SD* = 1.5).

### Measures

All measures used in Study 2 were presented in Mandarin Chinese, with all items translated from English following Brislin’s (1986) translation-back translation procedure. Unless otherwise specified, all measures in Study 2 used a five-point Likert-type scale ranging from 1 = “Strongly disagree” to 5 = “Strongly agree.” All items are presented in Appendix A.

#### Employee boundary blurring behavior (T1)

Supervisors rated employee boundary blurring behavior with the same four-item scale used in Study 1 (*α* = 0.83).

#### Supervisor liking toward the employee (T2)

Supervisors rated their liking for employees using the same four-item scale as in Study 1 (*α* = 0.97).

#### Abusive supervision (T3)

Employees rated how frequently their supervisor engages in abusive supervision with the same five-item scale as in Study 1 (1 = “Never”; 5 = “Very often”; *α* = 0.88).

#### Control variables

We controlled for demographic variables previously found to relate to supervisor liking toward the employee and/or abusive supervision (Bernerth and Aguinis, 2016). Specifically, we controlled for supervisor gender (female = 0; male = 1), age (in years), and education (in years). We controlled for supervisor gender because women are more concerned about interpersonal relationships than men, more likely to show liking to others, and less likely to engage in abusive supervision (Collins and Miller, 1994; Burton and Hoobler, 2006). Supervisor age was controlled for because elderly supervisors are less aggressive and less likely to engage in abusive supervision (Barling et al., 2009). We controlled for supervisors’ education because those with higher levels of formal education have been found less likely to abuse employees (Eesley and Meglich, 2013). Besides these demographic characteristics, we also controlled for dyadic tenure because it shapes the quality of social exchanges between supervisors and employees (Cogliser and Schriesheim, 2000; Maslyn and Uhl-Bien, 2001; Erdogan and Liden, 2002), which may influence abusive supervision. It should be noted that excluding all control variables did not affect the significance of our findings.

### Analytic strategy

As our data were nested by supervisor, we employed the “two-level COMPLEX” function in Mplus to test our hypotheses with 10,000-resample bootstrapping, which considering stratification, non-independence of observations due to cluster sampling (Muthén and Muthén, 2017). We used grand-mean centering for all explanatory variables before entering them into the regression model (Hofmann and Gavin, 1998). As in Study 1, we tested the indirect effect using RMediation.

## Study 2 results

Descriptive statistics and correlations are reported in Table 3. To provide further evidence supporting the factor structure and discriminant validity of our measures, we conducted confirmatory factor analyses (CFAs) involving our three key constructs (i.e., employee boundary blurring behavior, supervisor liking toward the employee, and abusive supervision). Results revealed that the three-factor structure had a good fit to the data (*χ^2^*(62) = 314.41, *p* < 0.001; Standardized Root Mean Squared Residual [SRMR] = 0.05, Comparative Fit Index [CFI] = 0.89, Tucker Lewis Index [TLI] = 0.87; Hu and Bentler, 1999) and fitted better than either of the two-factor models (combining employee boundary blurring behavior with supervisor liking toward the employee; *χ^2^*(64) = 606.72, *p* < 0.001; SRMR = 0.13, CFI = 0.77, TLI = 0.72; combining supervisor liking toward the employee with abusive supervision; *χ^2^*(64) = 886.48, *p* < 0.001; SRMR = 0.19, CFI = 0.65, TLI = 0.58). Based on these results, we proceeded to hypothesis testing with the hypothesized three-factor model.

**Table 3:** Descriptive statistics and correlation coefficients of variables in Study 2.

Variables	Mean	*SD*	1	2	3	4	5	6
1. Supervisor gender	0.82	0.39						
2. Supervisor age	32.35	7.72	0.19^**^					
3. Supervisor education	11.57	2.76	0.06	0.14^*^				
4. Dyadic tenure	1.97	1.54	0.02	0.08	−0.03			
5. Employee boundary blurring behavior (T1)	2.66	0.90	0.25^***^	0.12	0.22^**^	0.10		
6. Supervisor liking toward the employee (T2)	4.17	0.74	0.04	−0.13	−0.19^**^	−0.01	0.29^***^	
7. Abuse Supervision (T3)	1.21	0.43	0.09	0.03	0.15^*^	0.03	0.04	−0.17^*^

*n* = 216 at individual level, *n* = 49 at team level. T1/2/3 = Time 1/2/3. For supervisor gender, 0 = female, 1 = male.

^*^p < 0.05; ^**^p < 0.01; ^***^p < 0.001.

### Tests of hypotheses

The hypothesis testing results are reported in Table 4. Hypothesis 1 proposes that employee boundary blurring behavior is positively related to supervisor liking toward the employee. The results revealed that employee boundary blurring behavior was significantly positively related to supervisor liking toward the employee (*b* = 0.29, *SE* = 0.12, *p* = 0.02). Thus, Hypothesis 1 was supported in Study 2.

**Table 4:** The effects of employee boundary blurring behavior and supervisor liking toward the employee on abusive supervision in Study 2.

Variables	Supervisor liking toward the employee (T2)						Abusive supervision (T3)					
	Model 1			Model 2			Model 3			Model 4		
	*b*	*SE*	*t*	*b*	*SE*	*t*	*b*	*SE*	*t*	*b*	*SE*	*t*
Supervisor gender	0.14	0.13	1.13	0.08	0.14	0.53	0.09	0.06	1.40	0.10	0.07	1.46
Supervisor age	−0.13	0.11	−1.19	−0.14	0.10	−1.45	−0.01	0.08	−0.13	−0.03	0.09	−0.40
Supervisor education	−0.17	0.11	−1.49	−0.23	0.11	−2.06^*^	0.15	0.08	1.91	0.12	0.09	1.34
Dyadic tenure	−0.04	0.06	−0.61	−0.06	0.06	−1.08	0.03	0.08	0.42	0.02	0.08	0.28
Employee boundary blurring behavior (T1)				0.29	0.12	2.44^*^	−0.01	0.08	−0.17	0.03	0.08	0.41
Supervisor liking toward the employee (T2)										−0.16	0.07	−2.27^*^
Constant	5.63	0.35	16.18^***^	5.63	0.34	16.55^***^	2.81	0.25	11.16^***^	3.73	0.45	8.28^***^
*R^2^*	0.06			0.14			0.03			0.05		

*n* = 216 at individual level, *n* = 49 at team level. T1/2/3 = Time 1/2/3. For supervisor gender: 0 = female, 1 = male.

^*^p < 0.05; ^**^p < 0.01; ^***^p < 0.001.

Hypothesis 2 proposes that supervisor liking toward the employee is negatively related to abusive supervision. As shown in Model 4 of Table 4, supervisor liking toward the employee was significantly negatively related to abusive supervision (*b* = −0.16, *SE* = 0.07, *p* = 0.02). Thus, Hypothesis 2 was supported in Study 2.

Hypothesis 3 proposes that employee boundary blurring behavior has a positive indirect effect on abusive supervision *via* supervisor liking toward the employee. RMediation was used to test the indirect effect by multiplying the path coefficient from employee boundary blurring behavior to supervisor liking toward the employee with the path coefficient from supervisor liking toward the employee to abusive supervision. The results revealed that the indirect effect was significantly negative (estimate = −0.05, *SE* = 0.03, 95% CI = −0.10, −0.01). Thus, Hypothesis 3 was supported in Study 2.

## General discussion

Abusive supervision has long been found to have remarkably negative impacts on individual and organizational outcomes (Priesemuth et al., 2014; Barnes et al., 2015; Tepper et al., 2017; Wee et al., 2017; Ju et al., 2019; Liao et al., 2021). While researchers have shown growing interest in identifying what factors contribute to reducing abusive supervision (e.g., Restubog et al., 2011; Liu et al., 2012; Mawritz et al., 2012; Barnes et al., 2015), few studies have adopted a bottom-up perspective to explore whether employees can deploy certain behavioral strategies to reduce supervisors’ abuse. In this research, we draw on self-disclosure theory (Cozby, 1973; Collins and Miller, 1994) to develop our theoretical model explaining whether and how employee boundary blurring behavior affects abusive supervision. Findings from a scenario-based experiment and a multi-source, multi-wave survey revealed that supervisors tend to like employees who engage in boundary blurring behavior and, consequently, reduce the extent of abusive supervision directed toward them.

### Implications for theory

Our research makes several theoretical contributions to abusive supervision literature and boundary management literature. First, we extend the abusive supervision literature by demonstrating the potential effectiveness of a bottom-up behavioral strategy (i.e., boundary blurring behavior) to shield employees from abusive supervision. Prior studies on the antecedents of abusive supervision have primarily focus on contextual and supervisory predictors, such as culture, organizational norm, and supervisors’ stress (e.g., Kiazad et al., 2010; Restubog et al., 2011; Tepper et al., 2011; Liu et al., 2012; Mawritz et al., 2012; Barnes et al., 2015). These studies have, however, largely overlooked another important research perspective, namely a bottom-up perspective that explores how employees can engage in proactive behaviors to avoid being abused. Thus, we contribute to the abusive supervision literature by addressing an overlooked but fundamental antecedent—employee boundary blurring behavior—which reduces the likelihood for employees to be abused by their supervisors. By exploring whether employee boundary blurring behavior reduces abusive supervision, our findings help to gain a more complete understanding of antecedents of abusive supervision and provide theoretical guidance for employees to avoid being abused.

Second, we enrich the abusive supervision literature by unfolding the mechanism through which non-work-related interactions between employees and supervisors (i.e., employee boundary blurring behavior) influence abusive supervision. Drawing on self-disclosure theory (Cozby, 1973; Collins and Miller, 1994), we suggest that employee boundary blurring behavior increases supervisor liking toward them, leading to a close, high-quality supervisor-employee relationship in which abusive supervision becomes less likely. By examining the mediating mechanism, our research deepens understanding of how to reduce abusive supervision through boundary blurring behavior.

Third, we extend the boundary management literature by exploring whether employees’ blurring of the personal-professional boundary can positively influence the supervisor-employee relationship. Prior studies of boundary management have mainly focused on explaining work–family conflict and balance by investigating how employees manage the boundaries between work and family roles (Ashforth et al., 2000; Kreiner et al., 2009; Allen et al., 2014). However, these studies have overlooked the effect of employees’ boundary management on workplace interactions (e.g., interactions between employees and their supervisors). Unlike most boundary management research, we explored how blurring the boundary between professional and personal domains can potentially benefit workplace interactions. Our work thus enriches this literature by identifying the potential positive outcomes of boundary blurring behavior in the supervisor-employee relationship.

### Implications for practice

Our research has several important managerial implications. First, our findings suggest that employee boundary blurring is an effective strategy to protect employees from abuse by their supervisors. Therefore, we encourage employees to engage in more boundary blurring behaviors when interacting with supervisors, which will make supervisors view them more favorably, thus minimizing the risk of being abused. Employees could, for example, share personal information with their supervisors, communicate with them more often on social media, or engage in non-work-related activities with them. Also, organizations could provide more opportunities for supervisors and employees to establish close informal working relationships, allowing supervisors and employees to interact more outside work. For example, organizations could hold birthday parties, group dinners, sporting events, and other activities to encourage non-work-related social interaction among supervisors and employees.

Second, our findings reveal that employee boundary blurring behavior reduces abusive supervision *via* supervisor liking for the employee. This outcome emphasizes the importance of cultivating supervisor liking for employees as a means to reduce abusive supervision. Therefore, we encourage employees to establish high-quality relationships with their supervisors by engaging in boundary blurring behaviors, thereby making supervisors more inclined to like them. Additionally, supervisors need to recognize that increasing their liking for employees, especially those toward whom they hold negative attitudes, can help to reduce their own abusive supervision.

### Strengths, limitations, and future directions

This study has several strengths: in particular, it employs a mixed design that combines an experiment with a multi-source, multi-wave field study, using samples from both America and China. However, it also has several limitations that could be addressed in future research. First, the causal effect of employee boundary blurring behavior on abusive supervision was inferred from a scenario-based experiment, rather than a real organizational context. Although scenario-based experiments in which participants act as supervisors are often used in organizational behavior research (Lam et al., 2017; Qin et al., 2018b; Yeung and Shen, 2019), participants in Study 1 may act differently in a real managerial context. Therefore, we encourage future studies to replicate our model using a field experimental design. For example, management scholars can cooperate with a company and randomly assign its employees into two group: for experimental group, researchers can instruct them to perform more boundary blurring behaviors with supervisors; for control group, researchers can instruct them to maintain the previous interactive ways with supervisors. In this way, future studies can test the causal effect of employee boundary blurring behavior on abusive supervision in a real organizational context.

Second, we did not examine the boundary conditions of the indirect effect of employee boundary blurring behavior on abusive supervision. This omission limits our understanding of whether the effect is contingent on some individual or contextual factors. In future research, it is imperative to identify any moderators of the effect of employee boundary blurring behavior on abusive supervision. For example, one study suggests that supervisors with integrating (rather than segmenting) boundary management preferences show a higher liking toward employees who engage in boundary blurring behavior (Ashforth et al., 2000). Therefore, the negative relationship between boundary blurring behavior and abusive supervision could be moderated by the supervisor’s boundary management preferences.

Third, regarding employee boundary blurring as a strategy to reduce abusive supervision, previous research suggests that outcomes may differ between active and reactive behaviors (Harari et al., 2021). Compared to reactive employee boundary blurring behavior, active boundary blurring may become a burden that embarrasses supervisors and results in other undesirable consequences. Therefore, we encourage further research to explore the effects of different types of boundary blurring (i.e., active or reactive) on abusive supervision.

## Conclusion

Given the severe negative impacts of abusive supervision, a series of studies have explored how to reduce abusive supervision by identifying the contextual and supervisory predictors of abusive supervision. However, we still have relatively limited knowledge about the antecedents of abusive supervision from the employee’s perspective. Based on self-disclosure theory, this research explores the effect of a bottom-up behavioral strategy (i.e., employee boundary blurring behavior) for employees to reduce or avoid being abused. Specifically, we posit that employee boundary blurring behavior will decrease abusive supervision *via* supervisor liking toward the employee. Because supervisors may perceive that employees who engage in boundary blurring behavior as more enthusiastic and desire to build a more intimate relationship with them. Therefore, by engaging in boundary blurring behavior, employees may enhance supervisor liking toward them and avoid being abused. Findings from a scenario-based experiment and a multi-source, multi-wave survey supported our hypotheses. In sum, we hope our findings will encourage future studies to identify more effective and safe solutions for employees to minimize the risk of being abused by supervisors.

## Data availability statement

The raw data supporting the conclusions of this article will be made available by the authors, without undue reservation.

## Ethics statement

The studies involving human participants were reviewed and approved by the Declaration of Helsinki. The patients/participants provided their written informed consent to participate in this study.

## Author contributions

LJ and XQ formulated the research idea. LJ, GH, LY, and XQ, designed and conducted the study. LJ and LY analyzed the data. All authors contributed to the article and approved the submitted version.

## Funding

This research was funded by a Fulbright Scholarship, sponsored by the U.S. government, and two grants funded by National Natural Science Foundation of China (Grant No. 72272155 and 71872190), awarded to XQ; China Postdoctoral Science Foundation (Grant No. 2022 M713555), and Tongshan Foundation for Young Scholars of Humanities and Social Sciences at Sun Yat-Sen University (Grant No. 2022066), awarded to GH.

## Conflict of interest

The authors declare that the research was conducted in the absence of any commercial or financial relationships that could be construed as a potential conflict of interest.

## Publisher’s note

All claims expressed in this article are solely those of the authors and do not necessarily represent those of their affiliated organizations, or those of the publisher, the editors and the reviewers. Any product that may be evaluated in this article, or claim that may be made by its manufacturer, is not guaranteed or endorsed by the publisher.

## Appendix A

### Scale items used in Study 1 and Study 2

#### Employee boundary blurring behavior

1. This employee would like to connect with me on social mediate (e.g., Facebook, WeChat).
2. This employee would like to talk with me about his/her personal life during work hours.
3. This employee would like to go to company-sponsored social activities with me (e.g., holiday parties, company picnics, sporting events).
4. This employee would like to go to employee-initiated social events with me (e.g., drinks after work, lunch, golf).

#### Supervisor liking toward the employee

1. This employee seems like a pleasant person to me.
2. I think it is pleasant to work with this employee.
3. This employee fits well in my team.
4. I would like to work with this employee.

#### Abusive supervision

1. To ridicule him/her.
2. To tell him/her that his/her thoughts or feelings are stupid.
3. To put him/her down in front of others.
4. To make negative comments about him/her to others.
5. To tell him/her that he/she is incompetent.
